# The correlation between stable angina and inflammatory factors and blood lipids: a case-control study

**DOI:** 10.3389/fcvm.2024.1443450

**Published:** 2024-10-23

**Authors:** Lei Xiang

**Affiliations:** Department of Cardiovascular Medicine, Fifth Affiliated Hospital of Sun Yat-sen University, Zhuhai, China

**Keywords:** stable angina, inflammatory factors, blood lipids, prognosis, a case-control study

## Abstract

**Objective:**

In this study, we aimed to compare the levels of inflammatory markers (C-reactive protein, CRP; procalcitonin, PCT) and blood lipids (total cholesterol, TC; triglyceride, TG; high-density lipoprotein cholesterol, HDL-C; low-density lipoprotein cholesterol, LDL-C) between patients with stable angina and control group, and to explore the correlation between these parameters and the severity and prognosis of stable angina.

**Methods:**

We retrospectively selected 113 patients with stable angina and 128 control group from the medical record system, and compared their inflammatory factors and blood lipids. We also assessed the severity of angina using the Canadian Cardiovascular Society (CCS) classification and followed up the patients for 1 year to record any cardiovascular events.

**Results:**

We found that patients with stable angina had significantly higher levels of CRP, TC, TG, and LDL-C, and lower levels of HDL-C than control group. Moreover, CRP, TC, TG, and LDL-C were positively correlated with the severity of angina, while HDL-C was negatively correlated. During the follow-up period, 37 patients with stable angina experienced cardiovascular events, and they had higher levels of CRP, TC, TG, and LDL-C, and lower levels of HDL-C than those who did not.

**Conclusion:**

Our study suggests that inflammation and dyslipidemia are closely related to stable angina, and that inflammatory factors and blood lipids can be used as indicators of the severity and prognosis of stable angina.

## Introduction

Stable angina is a clinical syndrome characterized by chest pain or discomfort that occurs when the myocardial oxygen demand exceeds the supply, usually due to coronary artery stenosis (1–3). Stable angina is a common manifestation of chronic coronary syndrome, which affects about 100 million people worldwide and is associated with increased morbidity and mortality (4). The main cause of stable angina is atherosclerosis, a chronic inflammatory disease of the arterial wall that leads to plaque formation and luminal narrowing (5). Inflammation and dyslipidemia are two important factors that contribute to the initiation and progression of atherosclerosis. Inflammation is involved in all stages of atherosclerosis, from endothelial dysfunction to plaque rupture and thrombosis (6, 7). Dyslipidemia, especially elevated levels of low-density lipoprotein cholesterol (LDL-C) and reduced levels of high-density lipoprotein cholesterol (HDL-C), promotes the accumulation of lipid-laden macrophages in the arterial wall, resulting in foam cell formation and plaque growth (8, 9). Therefore, inflammation and dyslipidemia are potential targets for the prevention and treatment of stable angina and its complications.

Several studies have investigated the relationship between stable angina and inflammatory factors and blood lipids, but the results are inconsistent and controversial. Some studies have reported that patients with stable angina have higher levels of inflammatory markers, such as C-reactive protein (CRP) or procalcitonin (PCT), than control group (10), while others only supported high-sensitivity CRP (11). Similarly, some studies have shown that patients with stable angina have higher levels of total cholesterol (TC), triglyceride (TG), and LDL-C, or lower levels of HDL-C than control group (12, 13), while others did not observe this association (14). Moreover, the correlation between inflammatory factors and blood lipids and the severity and prognosis of stable angina is not well established. Therefore, there is a need for further research to clarify the role of inflammation and dyslipidemia in stable angina.

In this study, we aimed to compare the levels of inflammatory factors (CRP, PCT) and blood lipids (TC, TG, HDL-C, LDL-C) between patients with stable angina and control group, and to explore the correlation between these parameters and the severity and prognosis of stable angina. We hypothesized that patients with stable angina would have higher levels of inflammatory factors and blood lipids than control group, and that these parameters would be positively correlated with the severity and prognosis of stable angina.

## Methods

### Study design and population

We retrospectively selected the cases from the medical record system of the Fifth Affiliated Hospital of Sun Yat-sen University, from January 2020 to January 2022 and diagnosed them as stable angina based on coronary angiography and medical history. We retrospectively collected data from 113 patients with stable angina and 128 control group patients (no coronary stenosis or calcified spots confirmed by CTA or coronary angiography) from the medical record system. Inclusion criteria were as follows: (1) age between 40 and 80 years; (2) chest pain or discomfort that was provoked by exertion or emotional stress and relieved by rest or nitroglycerin; (3) no change in the frequency, duration, or intensity of angina episodes in the past 2 months; (4) no evidence of myocardial infarction on electrocardiogram (ECG) or cardiac biomarkers;(5) there were also lipid and inflammatory test results. The exclusion criteria were: (1) acute coronary syndrome; (2) unstable angina; (3) other causes of chest pain, such as pulmonary embolism, aortic dissection, or esophageal spasm; (4) severe heart failure; (5) severe renal or hepatic dysfunction; (6) malignancy; (7) autoimmune diseases; (8) inflammatory and infectious diseases; (9) missing clinical data; (10) use of anti-inflammatory or lipid regulator drugs, such as aspirin, diclofenac, statins, or corticosteroids, in the past month. The study protocol was approved by the ethics committee of the hospital, and the requirement for informed consent was waived due to the retrospective nature of the study.

### Data collection and measurement

We collected the demographic and clinical data of the participants, including age, sex, weight, height, smoking history, hypertension history, diabetes history, family history of coronary artery disease (CAD), history of acute myocardial infarction (AMI), history of percutaneous coronary intervention (PCI). We also assessed the severity of angina using the Canadian Cardiovascular Society (CCS) classification, which ranges from I to IV, with higher grades indicating more severe symptoms and limitations. We collected measured the blood pressure, heart rate, and body mass index (BMI) of the participants, and performed a 12-lead ECG. Blood samples for the analysis of inflammatory factors (CRP, PCT) and blood lipids (TC, TG, HDL-C, LDL-C) were obtained at the time of admission after overnight fasting, following routine clinical practice. We followed up the patients with stable angina for one year by telephone or outpatient visits, and recorded any cardiovascular events, such as angina worsening, acute myocardial infarction (AMI), Percutaneous coronary intervention (PCI), coronary artery bypass grafting (CABG), stroke, or death. The lost visit rate is 7%.

### Statistical analysis

We used SPSS software (version 26.0) for statistical analysis. We expressed the categorical variables as frequencies and percentages, and the continuous variables as means and standard deviations or medians and interquartile ranges, depending on their distribution. We compared the categorical variables between the two groups using chi-square test or Fisher's exact test, and the continuous variables using *t*-test or Mann-Whitney *U*-test. We performed Spearman's correlation analysis to examine the relationship between inflammatory factors and blood lipids and the severity of angina. We used Cox proportional hazards regression model to evaluate the association between inflammatory factors and blood lipids and the risk of cardiovascular events, adjusting for potential confounders. We considered a two-sided *p*-value of less than 0.05 as statistically significant.

## Results

### Baseline characteristics of the participants

The baseline characteristics of the participants are shown in Table 1. There were no significant differences in age and sex between the patients with stable angina and the control group. The patients with stable angina had higher BMI, blood pressure, and heart rate than the control group. The patients with stable angina also had higher prevalence of smoking, hypertension, diabetes, history of AMI, and history of PCI than the control group. However, both groups recorded zero cases for a family history of CAD.

**Table 1:** Baseline characteristics of the participants.

Variable	Stable angina (*n* = 113)	Control group (*n* = 128)	*p*-value
Age (years)	58.81 ± 8.42	57.99 ± 9.06	0.47
Sex (male,%)	73 (64.60)	71 (55.47)	0.15
BMI (kg/m^2^)	24.79 ± 3. 48	23.80 ± 3.30	0.02
Systolic blood pressure (mmHg)	138.24 ± 20.56	128.53 ± 18.86	<0.001
Diastolic blood pressure (mmHg)	84.59 ± 12.25	80.79 ± 12.43	0.02
Heart rate (bpm)	78.95 ± 13.85	75.78 ± 9.60	0.04
Smoking (%)	53 (46.90)	29 (22.66)	<0.001
Hypertension (%)	66 (58.41)	42 (32.81)	<0.001
Diabetes (%)	33 (29.20)	9 (7.03)	<0.001
Family history of CAD (%)	0	0	–
History of AMI (%)	3 (2.65)	0	0.10
History of PCI (%)	8 (7.08)	0	0.002
CCS grade (%)			<0.001
I	51 (45.13)	–	–
II	52 (46.02)	–	–
III	10 (8.85)	–	–
IV	0	–	–

### Comparison of inflammatory factors and blood lipids between the two groups

The levels of inflammatory factors and blood lipids between the patients with stable angina and the control group are shown in Table 2. The patients with stable angina had significantly higher levels of CRP, TC, TG, and LDL-C, and lower levels of HDL-C than the control group. There was no significant difference in PCT levels between the two groups.

**Table 2:** Comparison of inflammatory factors and blood lipids between the two groups.

Variable	Stable angina (*n* = 113)	Control group (*n* = 128)	*p*-value
CRP (mg/L)	1.32 (0.40–3.37)	0.20 (0.10–0.60)	<0.001
PCT (ng/ml)	0.10 (0.10–0.10)	0.10 (0.10–0.10)	0.23
TC (mmol/L)	4.62 ± 1.00	4.22 ± 0.78	0.001
TG (mmol/L)	1.89 ± 1.50	1.47 ± 0.91	0.01
HDL-C (mmol/L)	1.08 ± 0.24	1.24 ± 0.40	<0.001
LDL-C (mmol/L)	2.80 ± 0.94	2.42 ± 0.71	<0.001

Continuous variables with normal distribution were presented as means and standard deviations, while others were presented as medians and interquartile ranges.

### Correlation between inflammatory factors and blood lipids and the severity of angina

**Figure 1:**
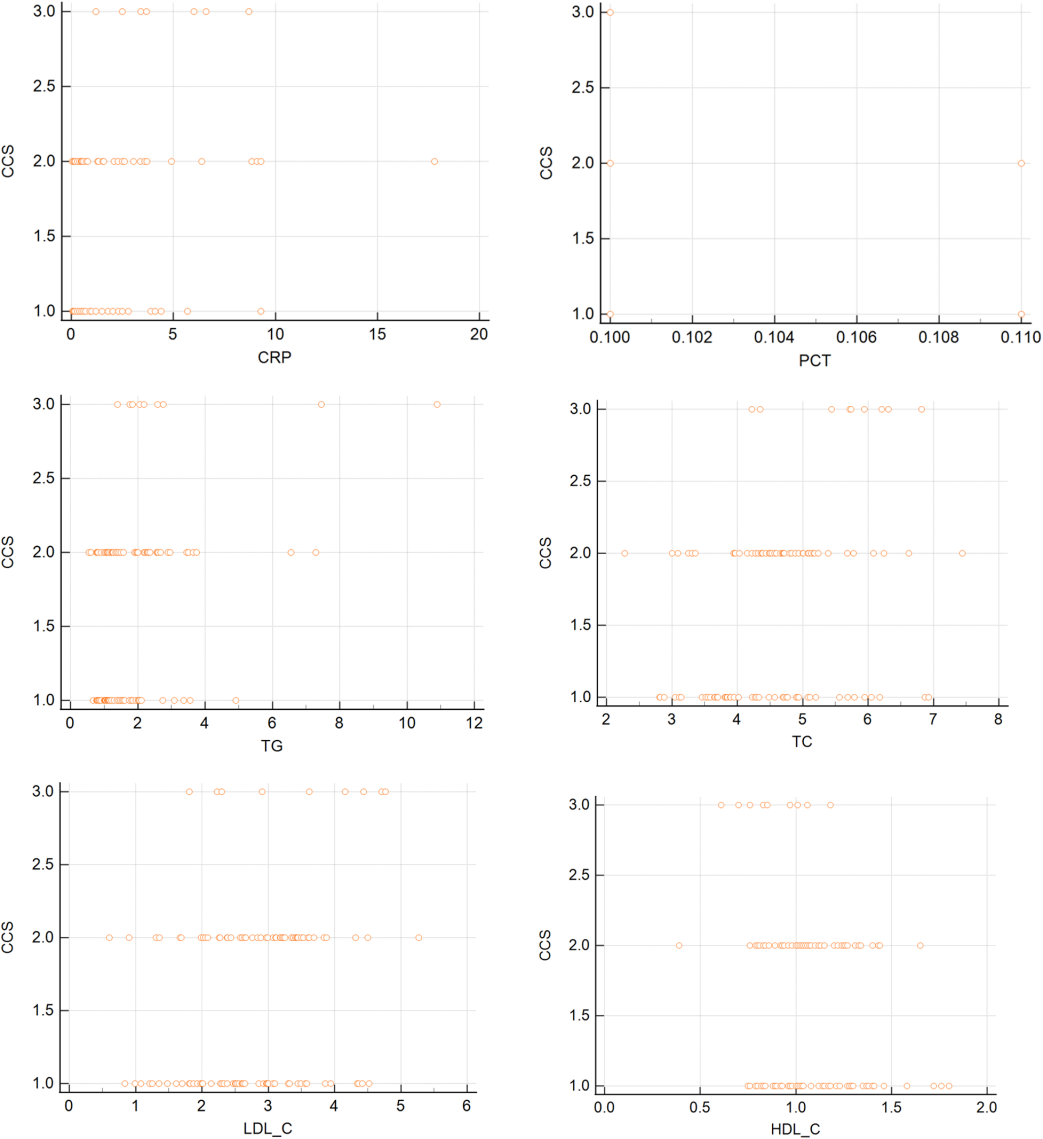
Scatter diagrams of inflammatory factors and blood lipids and the severity of angina.

We performed correlation analysis to examine the relationship between inflammatory factors and blood lipids and the severity of angina, as measured by the CCS grade. The results are shown in Table 3 and Figure 1. We found that CRP, TC, TG, and LDL-C were positively correlated with the CCS grade, indicating that higher levels of these parameters were associated with more severe angina symptoms and limitations. On the contrary, HDL-C was negatively correlated with the CCS grade, indicating that lower levels of this parameter were associated with more severe angina symptoms and limitations. There was no significant correlation between PCT and the CCS grade.

**Table 3:** Correlation between inflammatory factors and blood lipids and the severity of angina.

Variable	CCS grade	*p*-value
CRP (mg/L)	0.27	0.02
PCT (ng/ml)	0.01	0.97
TC (mmol/L)	0.31	0.001
TG (mmol/L)	0.33	0.001
HDL-C (mmol/L)	−0.21	0.03
LDL-C (mmol/L)	0.25	0.01

**Figure 2:**
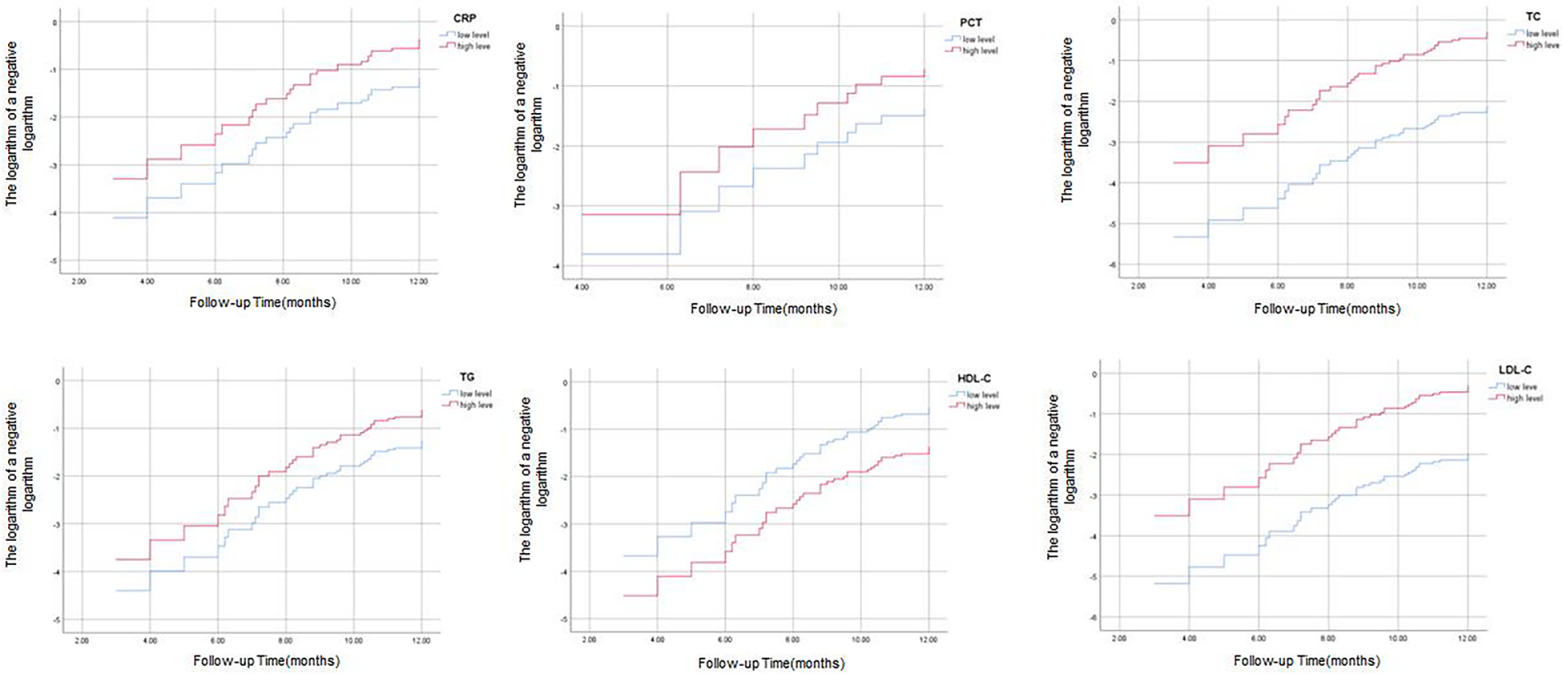
Cox regression analysis of PH proportional hazard to judge the results of CRP, PCT, TC, TG, HDL-C and LDL-C.

Association between inflammatory factors and blood lipids and the risk of cardiovascular events, patients with stable angina were recorded for any cardiovascular events that occurred during the follow-up period of the study. The incidence of cardiovascular events was 32.74% (37/113) in the patients with stable angina, and no cardiovascular events occurred in the control group. The cardiovascular events included angina worsening (*n* = 34), PCI (*n* = 3). No death occurred in either group. We used Cox proportional hazards regression model to evaluate the association between inflammatory factors and blood lipids and the risk of cardiovascular events, adjusting for age, sex, BMI, smoking, hypertension, diabetes, family history of CAD, history of AMI, history of PCI. We took the median as the critical point in the data of inflammatory factors and blood lipids and divided them into high level group and low level group. The results show that the two curves of CRP, TC, TG, LDL-C and LDL-H are roughly parallel, the effect of the factors examined on the survival risk is basically the same at different time points, and the survival risk of the two curves varies in proportion, and this risk ratio does not change with time, then the PH condition of Cox regression was valid, It is suggested that the basic assumptions have been passed, and it is appropriate to use Cox regression model for the data of this study in. While the two curves of PCT intersect, suggesting that the basic assumptions are not passed, and it is not appropriate for this study data to use Cox regression model (Figure 2). On this basis, we use univariate cox regression analysis to find that the *P* values of CRP, TC, TG, HDL-C and LDL-C are all less than 0.05 in Table 4, which can be included in multivariate cox regression analysis. The results are shown in Table 4, we found that CRP, TG, HDL-C, and LDL-C were independent predictors of cardiovascular events, with higher levels of these parameters indicating higher risk. HDL-C was also an independent predictor of cardiovascular events, with lower levels of this parameter indicating higher risk. TC was not a significant predictor of cardiovascular events.

**Table 4:** Univariate analysis and multivariate COX regression analysis of the relationship between inflammatory factors, blood lipids and the risk of cardiovascular events.

Variable	Univariate analysis		Multivariate analysis	
	Hazard ratio (95% confidence interval)	*p*-value	Hazard ratio (95% confidence interval)	*p*-value
CRP (mg/L)	1.09 (1.00–1.19)	0.04	1.18 (1.06–1.32)	0.01
TC (mmol/L)	2.07 (1.55–2.76)	<0.001	1.06 (0.53–2.11)	0.87
TG (mmol/L)	1.33 (1.15–1.54)	<0.001	1.30 (1.01–1.68)	0.04
HDL-C (mmol/L)	0.14 (0.03–0.64)	0.01	0.12 (0.02–0.93)	0.04
LDL-C (mmol/L)	2.41 (1.65–3.52)	<0.001	2.96 (1.36–6.47)	0.01

This table presents the hazard ratios (HR) with 95% confidence intervals (CI) and corresponding *p*-values for the relationship between selected inflammatory factors, blood lipid levels, and the risk of cardiovascular events among the study participants. The univariate analysis evaluates each variable independently, while the multivariate analysis adjusts for other factors in the model.

## Discussion

In this study, we compared the levels of inflammatory factors and blood lipids between patients with stable angina and control group, and explored the correlation between these parameters and the severity and prognosis of stable angina. We found that patients with stable angina had higher levels of CRP, TC, TG, and LDL-C, and lower levels of HDL-C than control group. Moreover, these parameters were associated with the severity of angina and the risk of cardiovascular events in patients with stable angina.

Compared to other studies (15, 16), the design of our research presents distinct differences that contribute to the observed findings. Firstly, unlike some studies that rely solely on cross-sectional data (15, 16), our study incorporated a 1-year follow-up to capture cardiovascular events, allowing for a more comprehensive analysis of prognosis. Secondly, we focused on a specific subset of inflammatory markers and blood lipids, while other studies (15, 16) may have included a broader range of biomarkers, potentially leading to varying conclusions. Additionally, we utilized the CCS classification to assess angina severity, providing a standardized measure that may not be consistently applied in other studies.

Our findings align with previous research that reported elevated levels of inflammatory markers and blood lipids in patients with stable angina compared to control groups (17, 18). These studies suggest that inflammation and dyslipidemia play critical roles in the pathophysiology of stable angina by promoting atherosclerotic plaque formation, impairing endothelial function, and disrupting vascular balance (19–21). The choice of our study method, including the use of CCS classification and targeted biomarkers, was driven by the aim to investigate specific and clinically relevant parameters that could aid in identifying high-risk patients and guiding management.

Our findings support the hypothesis that inflammatory factors and blood lipids are correlated with the severity and prognosis of stable angina. We observed that CRP, TC, TG, and LDL-C were positively correlated with the CCS grade, while HDL-C was negatively correlated. This implies that higher levels of these parameters reflect more severe angina symptoms and limitations, and may also indicate more extensive and complex coronary artery lesions (22–24). Furthermore, we found that CRP, TG, and LDL-C were independent predictors of cardiovascular events in patients with stable angina, while HDL-C was a protective factor. This suggests that higher levels of these parameters increase the risk of adverse outcomes, such as angina worsening, AMI, PCI, CABG, stroke, or death, in patients with stable angina, while lower levels of HDL-C decrease the risk. These results have demonstrated the prognostic value of inflammatory factors and blood lipids in patients with stable angina. These studies have proposed that inflammatory factors and blood lipids may serve as biomarkers for risk stratification and therapeutic intervention in patients with stable angina, and that regulating the levels of these parameters may improve the clinical outcomes of these patients.

This study introduces several novel insights that have significant implications for clinical practice. Unlike prior research (15, 16), which often focuses on broader patient populations or includes a wider range of biomarkers, our study specifically targets a well-defined group of patients with stable angina, providing a more focused and clinically relevant analysis. By integrating a 1-year follow-up for cardiovascular events and employing the CCS classification for angina severity, we offer a more precise assessment of the relationship between inflammatory factors, blood lipids, and cardiovascular risk. This study highlights the importance of early identification and management of these biomarkers to improve patient outcomes. The findings could potentially guide the development of targeted therapeutic strategies aimed at modulating these biomarkers, thereby reducing the risk of adverse cardiovascular events in patients with stable angina.

In this study, we observed no significant difference in PCT levels between patients with stable angina and the control group, despite PCT being known to respond more rapidly and reach maximum levels faster than CRP. One possible explanation for this finding is the timing of blood sample collection. Since this was a retrospective study, samples were collected according to routine clinical practice, typically at the time of admission. As stable angina is a chronic condition with potentially fluctuating levels of inflammatory markers, the timing of PCT measurement may have missed its peak levels, particularly if collected outside the window of maximum PCT expression. This timing issue highlights a limitation of our study and may partly explain the lack of significant difference in PCT levels.

However, our study has limitations that must be acknowledged. First, our sample size was relatively small, and the follow-up period was relatively short at only 1 year, which may limit the statistical power and the generalizability of our results. Additionally, we reported a 32.74% incidence of cardiovascular events among stable angina patients during this one-year follow-up period. It is important to note that this follow-up period may not fully capture the long-term outcomes or the progression of the disease, as serial studies were not conducted. Furthermore, due to the retrospective nature of this study, follow-up data were largely collected through telephone interviews rather than clinical visits. This approach limited our ability to obtain updated blood lipid and inflammatory marker levels after the cardiovascular events occurred. Therefore, we lack serial data to assess the potential changes in these parameters over time, which is a significant limitation in understanding the dynamic relationship between these markers and the long-term prognosis of stable angina patients. Second, we did not measure other inflammatory factors and blood lipids that may be relevant to stable angina, such as interleukin-6, tumor necrosis factor-alpha, lipoprotein(a), and apolipoprotein B. These biomarkers were not included because this study is a retrospective analysis based on routine clinical practice, where these specific markers are not commonly measured in stable angina patients. Third, we did not perform coronary angiography or other imaging modalities to assess the morphology and composition of the coronary plaques, which may provide more insights into the relationship between inflammation and dyslipidemia and stable angina. Fourth, we did not adjust for other potential confounders, such as patient compliance, diet, physical activity, stress, and environmental factors, that may influence the levels of inflammatory factors and blood lipids and the occurrence of cardiovascular events. Therefore, further studies with larger sample size, longer follow-up period, more comprehensive measurements, and more rigorous adjustments are needed to confirm and extend our findings.

## Conclusion

In conclusion, our study suggests that inflammation and dyslipidemia are closely related to stable angina, and that inflammatory factors and blood lipids can be used as indicators of the severity and prognosis of stable angina. Measuring the levels of these parameters may help to identify the high-risk patients and to guide the optimal management of stable angina. Lowering the levels of these parameters may improve the clinical outcomes of stable angina. Further studies are warranted to validate and elucidate the role of inflammation and dyslipidemia in stable angina.

## Data Availability

The original contributions presented in the study are included in the article/Supplementary Material, further inquiries can be directed to the corresponding author.
